# Large-Area Electrodeposited WSe_2_ over Graphene Electrodes for Optoelectronics

**DOI:** 10.1021/acsanm.4c07346

**Published:** 2025-05-16

**Authors:** Jiapei Zhang, Shibin Thomas, Ahmad Nizamuddin Muhammad Mustafa, Victoria Greenacre, Nikolay Zhelev, Syeda Ramsha Ali, Yisong Han, Shaokai Song, Hongwei Zhang, Aiden Graham, Nema M. Abdelazim, Sami Ramadan, Richard Beanland, Gillian Reid, Philip N. Bartlett, Kees de Groot, Yasir J. Noori

**Affiliations:** † School of Electronics and Computer Science, 7423 University of Southampton, Southampton SO17 1BJ, United Kingdom; ‡ School of Chemistry and Chemical Engineering, 7423 University of Southampton, Southampton SO17 1BJ, United Kingdom; § Department of Materials, 4615 Imperial College London, London SW7 2AZ, United Kingdom; ∥ Department of Physics, 2707 University of Warwick, Coventry CV4 7AL, United Kingdom; ⊥ FTKEK, Universiti Teknikal Malaysia Melaka, 76100 Malacca, Malaysia

**Keywords:** electrodeposition, 2D materials, TMD, tungsten diselenide, WSe_2_, graphene, electrode, heterostructure

## Abstract

Integrating graphene and transition metal dichalcogenides (TMDs) into layered material heterostructures brings together the exciting properties that each constituent 2D material offers. However, scaling the growth of graphene-TMD and related heterostructures remains a major challenge. In this work, we demonstrate the use of electrodeposition with a single source precursor (SSP), WSeCl_4_, to grow few-layer WSe_2_ using graphene as an electrode. Through characterization via photoluminescence, X-ray photoelectron, and Raman spectroscopy, we show that the electrodeposited WSe_2_ is stoichiometric and exhibits semiconducting and light-emitting properties. TEM imaging was also performed to show the ordering of the stacked layers of WSe_2_ over graphene, demonstrating the polycrystalline structure of WSe_2_. This work paves the way toward utilizing electrodeposition to stack multiple TMDs, including MoS_2_, WS_2_, and WSe_2_ over graphene for electronic and optoelectronic applications.

## Graphical abstract

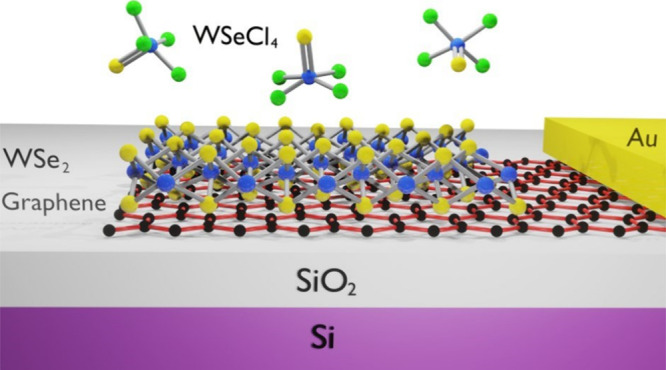

## 1 Introduction

Few-layer 2D transition metal dichalcogenides (TMDs) such as WSe_2_ have been used to make high-performance optoelectronic devices,^1–3^ due to their quantum efficiency that exceeds 70% and high carrier mobility.^4,5^ Moreover, most TMDs are n-type semiconductors as deposited, while WSe_2_ stands out because it is typically p-type and has been used in several demonstrations of p–n junction devices and transistors,^6^ making the incorporation of WSe_2_ with other n-type TMDs promising for complementary metal-oxide semiconductor electronics.^7^

On the other hand, graphene is a 2D material with zero band gap, which offers high conductivity.^8^ Therefore, graphene is widely used as an electrode material in a range of applications.^9^ Specifically, in the field of flexible and transparent electronics, such as field effect transistors (FETs), graphene’s high conductivity, high optical transparency, and stable chemical properties make it more interesting than electrode materials such as indium tin oxide (ITO).^10^ In addition to FETs, batteries,^11^ supercapacitors,^12^ and solar cells^13^ have also adopted graphene as device electrodes. Given the unique individual properties of WSe_2_ and graphene, integrating WSe_2_ with graphene in heterostructures is very promising for various electronic and optoelectronic applications. For example, FETs that consist of WSe_2_-graphene heterostructures can exhibit outstanding I_on/off_ ratios,^14^ and photodetectors with high responsivity have been fabricated using WSe_2_-graphene heterostructures.^15,16^

Exfoliation,^17,18^ chemical vapor deposition (CVD),^19–21^ and molecular beam epitaxy (MBE)^22–24^ are common methods for depositing WSe_2_ over graphene. Exfoliation can produce highly crystalline WSe_2_, but it has low scalability due to depositing size-limited flakes and the requirement of additional transfer steps. CVD is best known for growing WSe_2_ over SiO_2_, and a few reports have used it to grow WSe_2_ over graphene; however, the resultant films are typically discontinuous.^25^ Additionally, CVD is usually not an area-selective deposition method, which would require post-deposition lithography and etching steps to pattern the grown materials, potentially creating defects and contaminating the materials.^26^ In the case of MBE, the sticking coefficient of Se on the growth surface is low under ultrahigh vacuum conditions, impacting growth stoichiometry and limiting the choice of substrate.^27^

An alternative method that has seldom been explored is electrodeposition. It is a room temperature technique that lends itself to depositing materials at selective areas over conductive electrodes. Electrodeposition is carried out in an electrolyte solution containing the precursor to deliver the target material. Another distinct advantage of electrodeposition lies in its ability to function as a non-line-of-sight deposition technique, enabling the conformal coating of complex three-dimensional structures, including patterned structures with high aspect ratios. Compared with the high cost and harsh deposition environment of CVD and MBE, electrodeposition is a relatively low cost technique, and it is used routinely in the semiconductor industry for the deposition of magnetic films for hard drive read/write heads, and in the formation of Cu chip interconnects using the Damascene process.^28,29^ A few works have demonstrated the electrodeposition of tungsten diselenide (WSe_2_) over conducting oxide substrate using a dual source precursor system based on H_2_WO_4_ and SeO_2_.^30,31^ Single source precursors (SSP) contain both the metal and the chalcogen directly bonded in a molecular species and can offer a better-defined solution speciation in the electrolyte as well as a simpler voltammetric response from the electrolyte solution. This can be beneficial for the controlled growth of mono- and few-layer TMDs, where precise control of the deposition conditions is advantageous. We have demonstrated the use of SSPs for the electrodeposition of molybdenum disulfide (MoS_2_) in dichloromethane (CH_2_Cl_2_) using [N^n^Bu_4_]_2_[MoS_4_] ^32,33^ and tungsten disulfide (WS_2_) using [NEt_4_]_2_[WS_2_Cl_4_].^34,35^

Recently, we have also successfully electrodeposited WSe_2_ 2D layered thin films using WSeCl_4_ as an SSP.^36^ In this work, we exploit the same precursor to demonstrate the fabrication of a 2D heterostructure by electrodeposition of WSe_2_ over graphene. The presented results show, besides the novelty of graphene electrodes, an advancement in the material crystallinity and thickness control in comparison to other dual source precursor electrodeposited WSe_2_ films from previous reports.^30,31^ Using a variety of characterization techniques, we have shown that our WSe_2_ exhibits semiconductor properties, demonstrating the first light emission from an electrodeposited TMD film.

## 2 Experimental Section

The graphene electrodes were grown by CVD on a copper foil and transferred onto a SiO_2_/Si substrate using the wet transfer process.^37,38^ Thermal evaporation was used to deposit an Au film on part of the graphene film to make a solid contact pad to bias the graphene in the electrodeposition cell.^32^ The precursor preparation and electrodeposition experiment were all carried out in a glove box (Belle Technology, UK), which was circulated with N_2_ and maintained sub-10 ppm of O_2_ and H_2_O levels. The detailed synthesis of the SSP, WSeCl_4_, is given in a recent publication.^39^ The solvent acetonitrile (MeCN) (Fisher, 99.9%) was dried and degassed by refluxing with CaH_2_, followed by distillation. 0.1 M [Et_4_N]Cl (Sigma-Aldrich, ≥99.0%, dried in vacuo) was used as the supporting electrolyte. The electrodeposition was performed using a three-electrode electrochemical cell,^32^ where graphene was used as the working electrode, a Pt disc was used as a counter electrode, and an Ag/AgCl (in 0.1 M [Et_4_N]Cl in MeCN) was used as the reference electrode.

The sample thickness and roughness of WSe_2_ were confirmed by tapping mode atomic force microscopy (AFM) using a Park XE7 system. The Raman spectra were obtained from a Renishaw inVia Raman spectrometer by using a 532 nm wavelength laser at room temperature. Light excitation and collection were done via a 50× objective lens, which can reduce the laser spot diameter to 1 μm. The layered crystal structures of WSe_2_ were measured via a JEOL ARM200f double aberration-corrected transmission electron microscope (TEM) in bright and dark field mode using an acceleration voltage of 200 kV. The chemical state and stoichiometry were determined by X-ray photoelectron spectroscopy (XPS) using a Thermo Scientific Theta Probe and wavelength-dispersive X-ray spectroscopy (WDS) using an Oxford Instruments Wave Spectrometer that is coupled to a Hitachi SU70 scanning electron microscope at 10 kV. Photoluminescence Spectroscopy was measured using an FLS1000 photoluminescence spectrometer, where the material was excited via a 450 W continuous wavelength xenon lamp.

## 3 Results and Discussion

### 3.1 Electrochemistry of WSeCl_4_ on Graphene

Figure 1a shows a schematic image of the concept of WSe_2_ electrodeposition over graphene that is presented in this paper.

**Figure 1:**
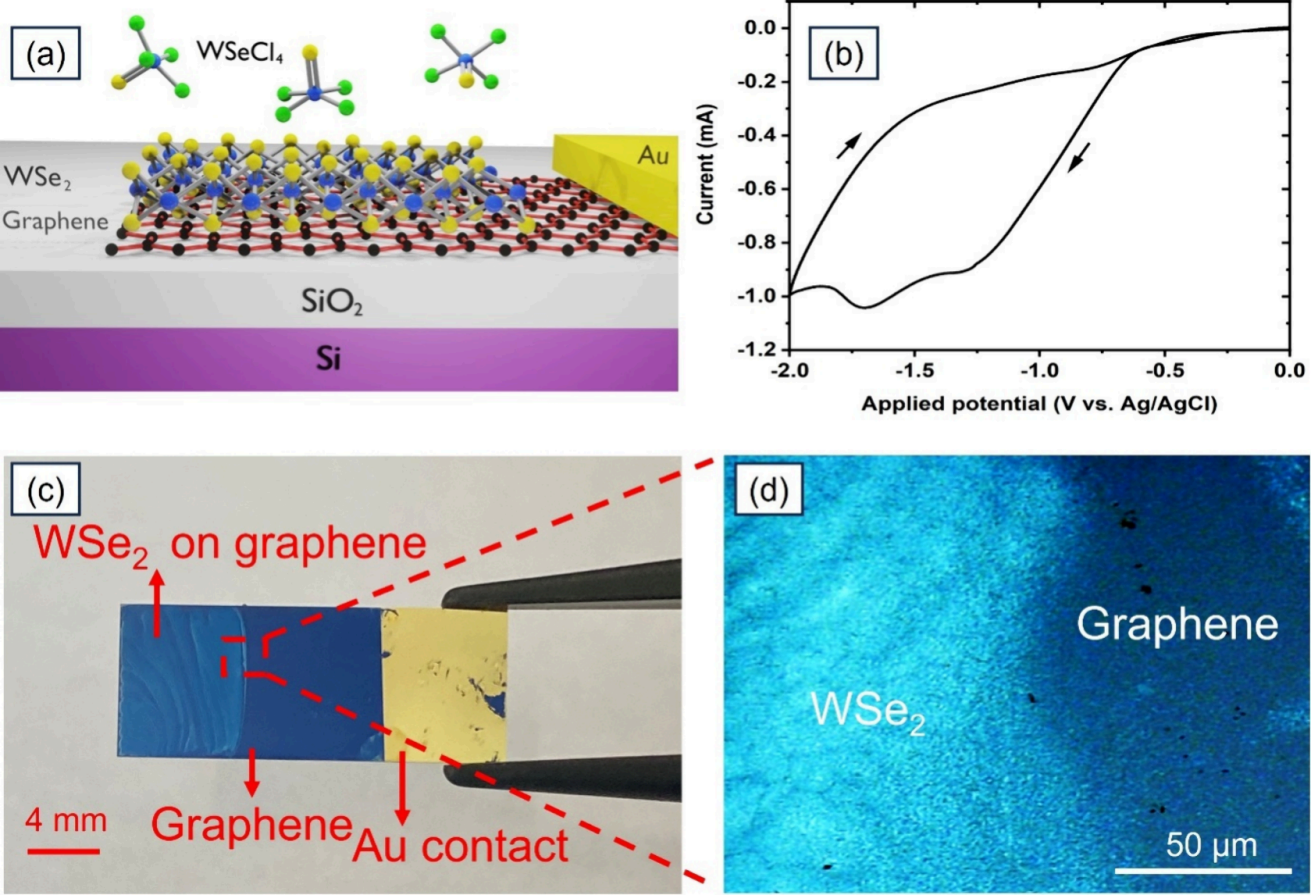
(a) Schematic illustration of the concept of this work showing WSe_2_ electrodeposited over graphene. (b) Cyclic voltammetry scans of the 10 mM WSeCl_4_ precursor in MeCN by using a graphene electrode. The scan rate is 50 mV s^–1^ and the arrows indicate the direction of potential scanning. (c) Photograph of the graphene/SiO_2_ substrate after 120 s electrodeposition, showing the area where the WSe_2_ was grown over graphene and a microscope image (d) of the WSe_2_ and graphene regions.

The electrochemical behavior of the [WSeCl_4_] precursor in conjunction with the graphene working electrode was first studied by conducting cyclic voltammetry (CV) scans as shown in Figure 1b. The electrochemical features in the CV on graphene are comparable to the CVs recorded on TiN and Pt electrodes, which are discussed in our recent report.^36^ The CV shows a clear dip in the current at around −1.4 V, which corresponds to the electroreduction of WSeCl_4_ to WSe_2_, and no anodic peaks are observed in the reverse scan. A plausible electrochemical reaction associated with the deposition of WSe_2_ is^36^

$$2[{\mathrm{WSeCl}}_{4}]+2e^{-}\to {\mathrm{WSe}}_{2}+{\mathrm{WCl}}_{6}+2{\mathrm{Cl}}^{-}$$ 1

Figure 1c shows a photograph of an as-deposited large-area WSe_2_ film (8 mm × 6 mm) after pulsed electrodeposition for 120 s (−1.4 V for 5 s followed by 0 V for 3 s, 24 cycles), demonstrating an obvious colour contrast to pristine graphene. Figure 1d depicts a microscope image of the deposited WSe_2_ film taken at the edge of the electrodeposition area, showing the uniform colour of the WSe_2_ film, to give an indication of the uniformity and continuity of the deposited material. After deposition, the films are annealed at 700 °C in N_2_ atmosphere in a furnace for 10 min to fully crystallize the WSe_2_ film.

### 3.2 AFM and Raman Spectroscopy

Atomic force microscope (AFM) characterization shows that the thickness of graphene is 2–3 nm, as shown by the green line in Figure 2a. Based on this measurement and optical microscope images of the graphene, we estimate that this represents a mono or bilayer graphene film.^40^ Although the AFM measured thickness of graphene is higher than their theoretical thickness for mono and bilayers, this is typically attributed to the graphene’s surface chemistry, such as the presence of solvent molecules above and below the graphene due to adsorption during the wet transfer process.^41,42^ Figure S1 shows that the 2D peak is symmetric (Figure S1a), and the ratio of *I*_2D_/*I*_G_ is higher than 1­(Figure S1b) which confirms the monolayer graphene.^43^ After taking into account the 3 nm thickness (green line in Figure 2d) obtained from the AFM on graphene electrodes, the 600 s deposition (−1.4 V for 5 s followed by 0 V for 3 s, 120 cycles) shows the actual as-deposited WSe_2_ thickness of around 37 ± 2 nm (blue line in Figure 2d), while the 120 s deposition exhibits an actual WSe_2_ thickness around 4 ± 1 nm (red line in Figure 2d), corresponding to around 4–7 layers of WSe_2_.^44^ The root mean square (RMS) roughness of the 120 s deposition is 2.0 nm, which confirms much better uniformity of the electrodeposited WSe_2_ films over graphene in comparison with previously reported electrodeposited WSe_2_ in the literature.^36^ However, further improvement to the uniformity is needed by reducing the side product formed during the electrodeposition, which has been discussed in our recent report on the electrochemistry of WSeCl_2_ precursor and the deposition of WSe_2_ on TiN.^36^ Moreover, the PMMA-assisted transfer process might also induce wrinkles in the graphene layer, which in turn affect the uniformity of electrodeposited WSe_2_ over graphene. In addition, any non-uniformity at the SiO_2_ surface can impact the adhesion and uniformity of both the transferred graphene and the subsequently electrodeposited WSe_2_. Optimisation of the transfer process of graphene and improvement of electrolyte composition and electrodeposition parameters can reduce wrinkles and byproducts.

**Figure 2:**
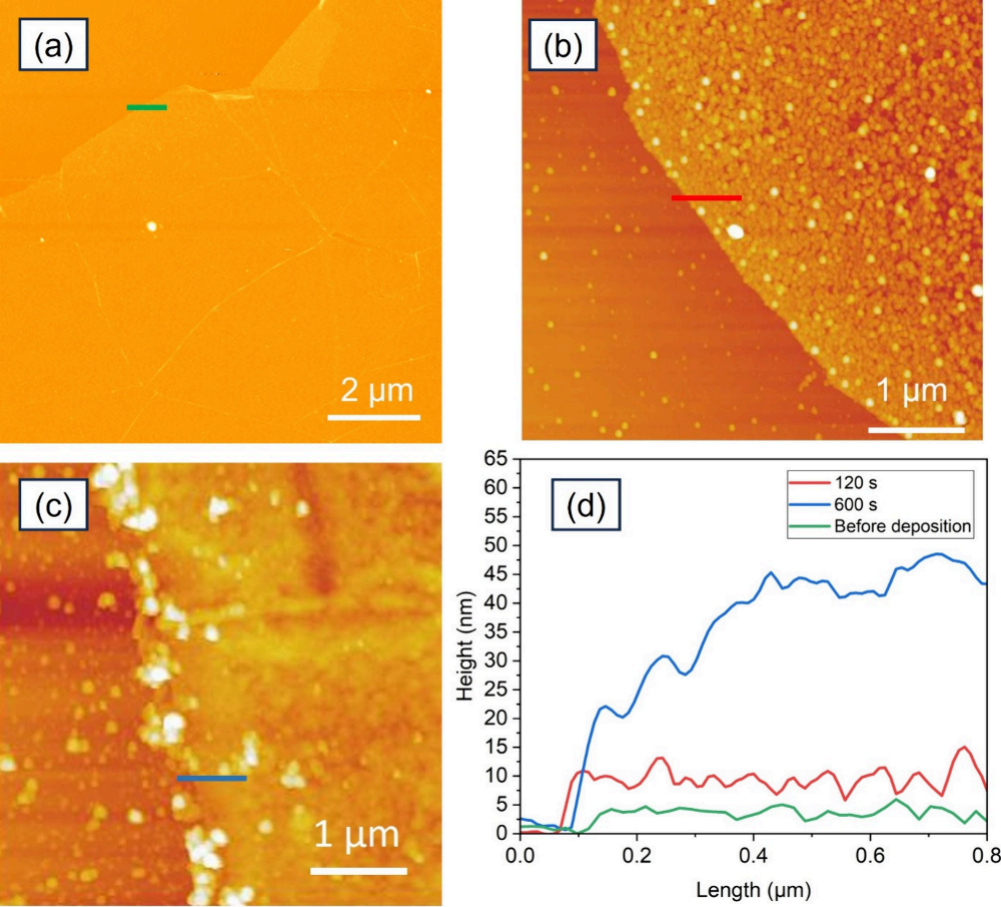
AFM topography images of WSe_2_ deposition on graphene for (a) before deposition, (b) 120 s, and (c) 600 s deposition. (d) Height profiles taken at the edges of the deposited films showing the total measured step heights.

The presence of WSe_2_ on the substrate, its uniformity and degree of crystallinity were investigated by measuring the Raman scattering of the film shown in Figure 3a. Figure 3b shows the Raman spectrum of electrodeposited WSe_2_, and that of a commercially grown WSe_2_ bulk crystal that was used here as a reference standard is shown in Figure S2a. It is clear that the E_2g_^1^ peak (249.5 cm^–1^) and A_1g_ peak (257.4 cm^–1^) are separated in the Raman spectrum of the reference sample, which is in accordance with the literature.^45^ However, there is no clear separation of these peaks in the Raman spectrum of electrodeposited WSe_2_, and only a major peak appears around 250 cm^–1^ which is also in accordance with the reference.^46^ The uniaxial strain resulting from interactions with the substrate, residual materials, or the electrodeposition process is responsible for the distinct separation of the Raman peaks in WSe_2_. From this standpoint, monolayers are significantly influenced by strain effects, whereas thicker flakes remain largely unaffected.^47^ Therefore, the reason for degeneracy of E_2g_^1^ and A_1g_ peak is that the presence of uniaxial strain is weak within the multilayer electrodeposited WSe_2_ which is confirmed by AFM results as shown before.

**Figure 3:**
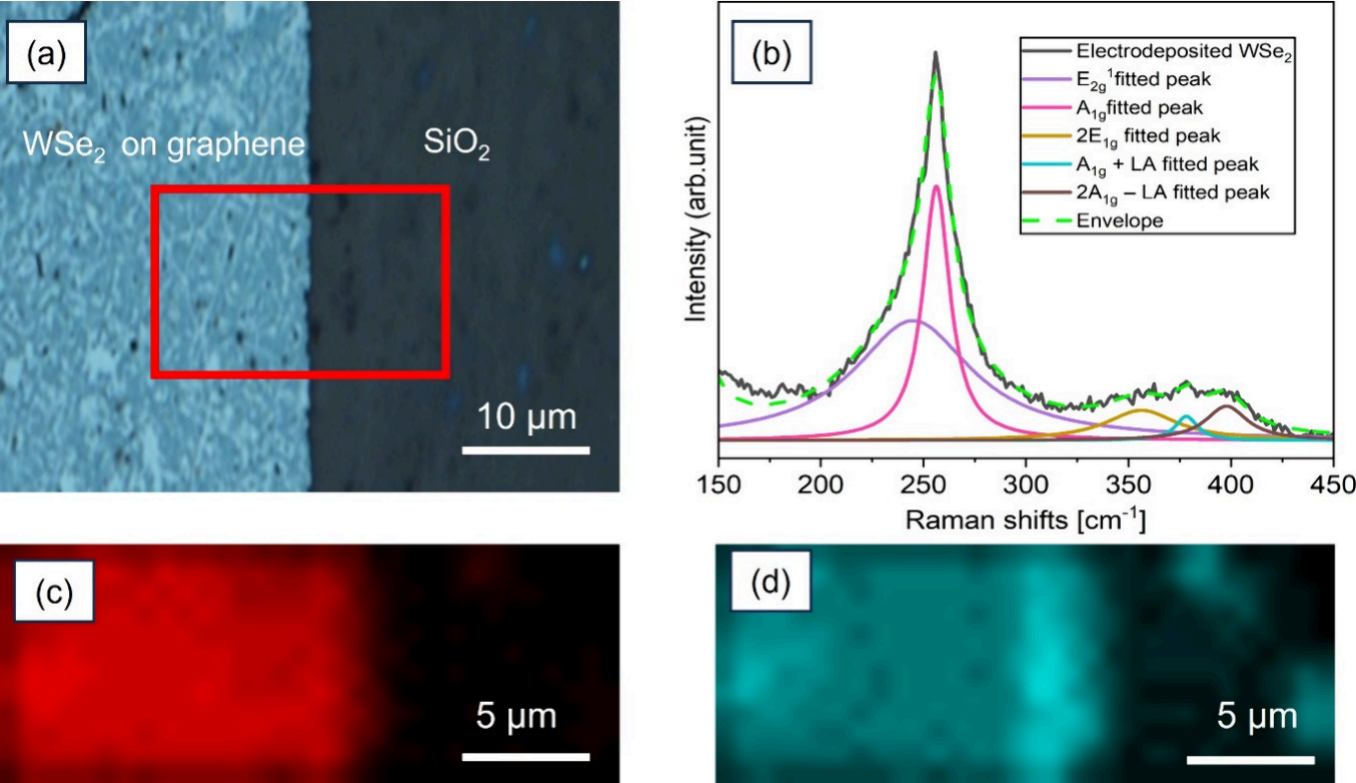
(a) Optical microscope image showing the contrast between the electrodeposited WSe_2_ (600 s) over the graphene area (left) and the underlying SiO_2_/Si substrate (right). The red frame indicates the Raman mapping area for (c, d). (b) Raman spectra taken using a 532 nm laser of an electrodeposited WSe_2_ film after annealing. Map plots for the Raman shift intensity taken at the signature peaks for (c) WSe_2_ (250 cm^–1^) and (d) graphene (1352 cm^–1^).

There are three minor peaks located between 350 and 400 cm^–1^ for both electrodeposited and commercial WSe_2_ crystals, which are attributed to second-order Raman features that arise from exciton-phonon coupling as reported by previous experimental and theoretical calculations.^48,46^ Figure 3c exhibits the WSe_2_ Raman mapping image which is plotted as a function of the intensity of the Raman peak at 250 cm^–1^. There is a significant color contrast between the electrodeposited WSe_2_ area (red) and SiO_2_/Si substrate (black), and the uniform map intensity across the electrodeposition area confirms the highly uniform film. The mapping image of graphene plotted as a function of the intensity of the Raman peak at 1352 cm^–1^ (D peak of graphene) is given in Figure 3d. The full range Raman spectrum of both WSe_2_ and graphene, and Raman mapping of the 2D peak are given in Figure S2b.

### 3.3 TEM, XPS, and WDS Characterization

TEM imaging was used to study the physical nature of the WSe_2_ film, including the crystallinity, domain size, and layer ordering. This is achieved by taking a lamella slice of the film using focused ion beam (FIB) milling. Pt and carbon were first deposited on the film as protection layers before the subsequent milling process. Figure 4a is the TEM image under bright field (BF) mode showing the C/Pt protection layers, WSe_2_, and SiO_2_/Si from top to bottom. In BF mode, WSe_2_ appears darker in comparison to other areas due to its constituent atoms being heavier than C, Si, and O, resulting in scattering more electrons, which are blocked in BF mode. A higher magnification image of the highlighted region in (a) is presented in Figure 4b. We found that the film consists of ordered crystal layers that extend horizontally along the substrate and stack vertically. Figure 4c shows a TEM image in dark field (DF) mode, where a highly scattered electron makes the heavy WSe_2_ film appear brighter. The layer-to-layer distance in the stack was measured to be 0.7 ± 0.1 nm, which matches with previously reported value.^44^ TEM images of the layered WSe_2_ indicate crystal domain sizes that range between 5 and 15 nm. Figure 4d is the BF-TEM image of the same region shown in Figure 4c, with an inset showing the WSe_2_ crystal under higher magnification, demonstrating its atomic structure. A guide to the eye is added showing the positions of the W (blue) and Se atoms (yellow).^49^

**Figure 4:**
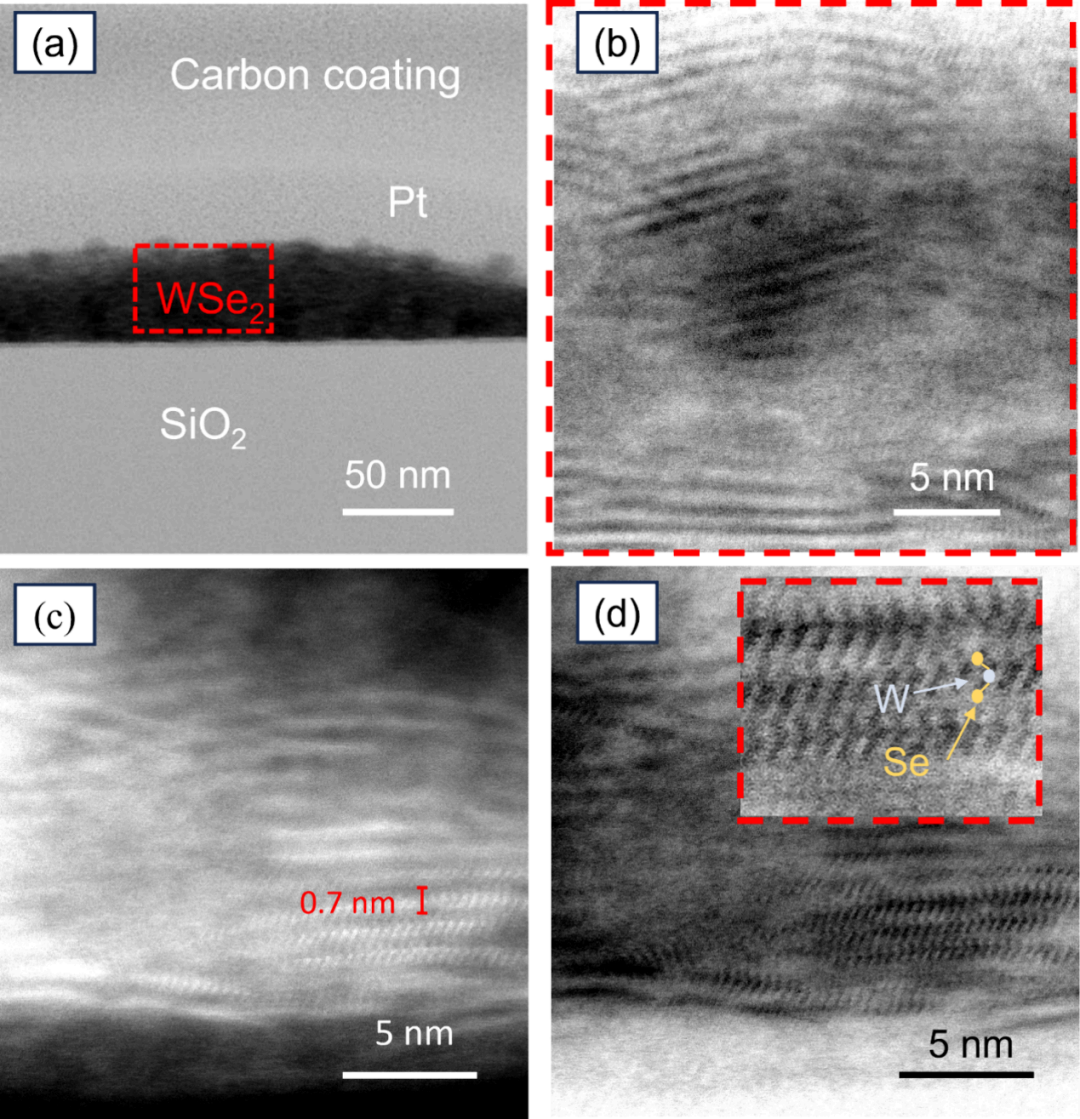
(a) Bright-field (BF) TEM image of electrodeposited WSe_2_ films (600 s). (b) High-magnification image of the highlighted region in (a) showing the layer ordering of the 2D WSe_2_ growing preferentially in the horizontal direction along the surface of the substrate. (c) Dark-field (DF) TEM images of electrodeposited films (d) BF-TEM image of as same region as (c) with the inset showing the layered crystal structure of WSe_2_ (blue: W atom, yellow: Se atoms).

The film composition was further investigated by XPS measurements. Figure 5a shows the XPS scan of the W atoms, where the two prominent peaks of 4f_7/2_ (32.43 eV) and 4f_5/2_ (34.53 eV) indicate the existence of WSe_2_.^50^ The other two significant peaks of 4f_7/2_ (35.78 eV) and 4f_5/2_ (37.93 eV) belong to WO_3_,^51^ which can be attributed to surface oxidation from the ambient atmosphere. As shown in Figure 5b, the binding energies of Se^2–^ 3d_5/2_ and Se^2–^ 3d_3/2_ are 54.6 and 55.4 eV, respectively, which confirms the existence of WSe_2_.^52–54^ The W:Se composition ratio in the film is found to be 1:1.83 based on the XPS measurement. This composition agrees with our previous work on the electrodeposition of thick WSe_2_ over TiN, which showed a W:Se composition ratio of 1:1.9 based on Energy Dispersive X-ray Spectroscopy (EDS).^36^ We were unable to obtain direct composition measurements from these WSe_2_ over graphene samples via EDS due to the large emission spectral overlap between W (Mα = 1.774 keV) and Si (Kα = 1.739 keV). We also performed wavelength dispersive X-ray spectroscopy (WDS) as shown in Figure S3 to obtain higher resolution spectroscopy measurements, but due to the small thickness of the film, the signal-to-noise ratio was too small to obtain quantitative data.

**Figure 5:**
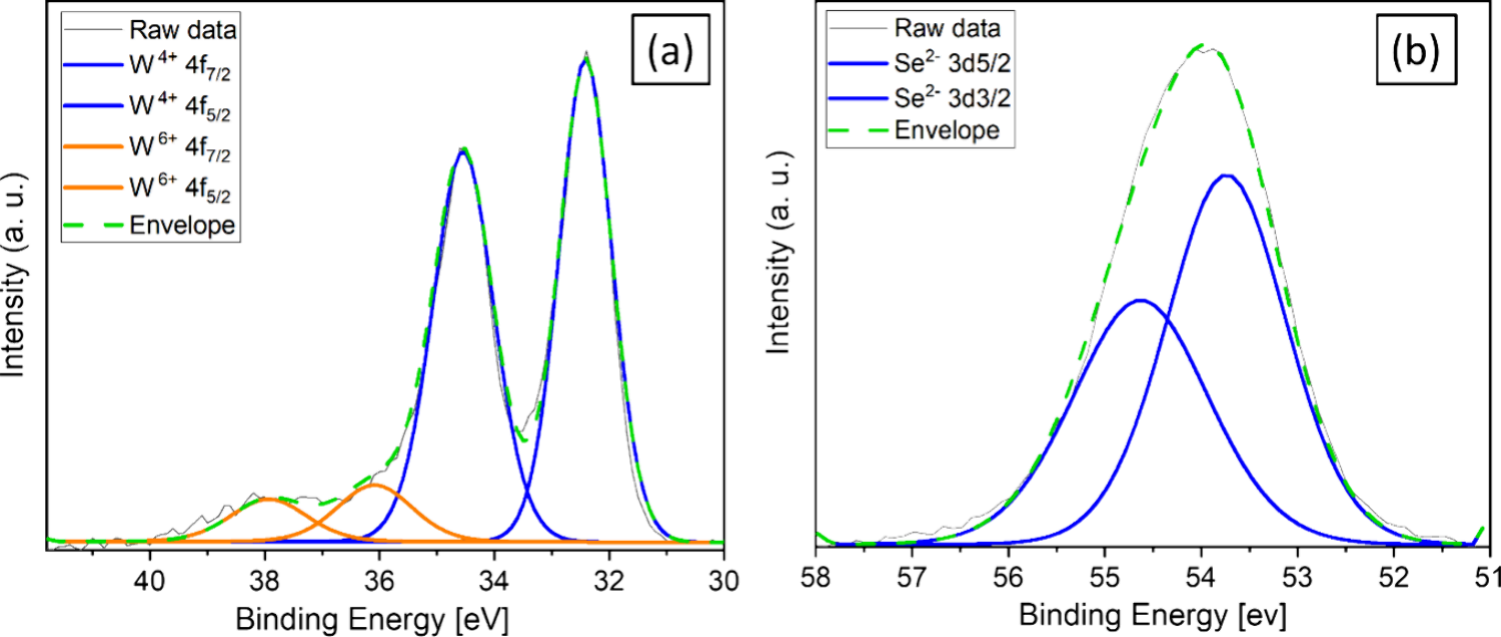
Large-area XPS measurements taken from the WSe_2_ over graphene film (600 s) at the binding energy ranges for (a) W and (b) Se.

### 3.4 Photoluminescence Spectroscopy

Photoluminescence (PL) spectroscopy has been adopted to investigate the optical properties of a material and to gain insight into its quality. Figure 6a displays the emission scans for graphene, electrodeposited WSe_2_ over TiN, and the electrodeposited WSe_2_ over the graphene layer. The graphene emission scan shows almost no PL emission, as expected. The emission scan of WSe_2_ on TiN and graphene displays a broad PL peak centred at 710 nm with a full-width-half-maximum of 108.7 ± 4.4 nm, depicting the bandgap of the material, which is one of the characteristics of WSe_2_. The noticeable broad emission suggests the presence of multiple excitonic transitions that result from an electronic state distribution. It should be noted that the typical PL peak of monolayer and bulk exfoliated WSe_2_ is located at around 750 nm (1.65 eV) and 900 nm (1.38 eV), respectively.^55–57^ It is unclear as to what causes the observed blue shift of the PL peak, but the behavior suggests a modification of the electronic band structure. Herein, we propose that the blue shift is influenced by substrate-induced strain, dielectric screening, and charge transfer interactions. Strain effects arise from lattice mismatch and thermal expansion differences between WSe_2_ and its substrate, particularly graphene and TiN. The resulting compressive strain modifies the band structure of WSe_2_, leading to an increase in its bandgap energy and a shift in PL emission toward higher energies. Previous reports have demonstrated similar bandgap modifications in strained WSe_2_ films.^58^ Additionally, dielectric screening plays a significant role in tuning the optical properties of WSe_2_. When placed on graphene, the surrounding electronic environment changes, which can reduce exciton binding energies and modify the emission energy.^59^ Another possible factor is charge transfer between WSe_2_ and graphene, which can induce band structure renormalization and lead to further shifts in the optical transition energies.^60^

**Figure 6:**
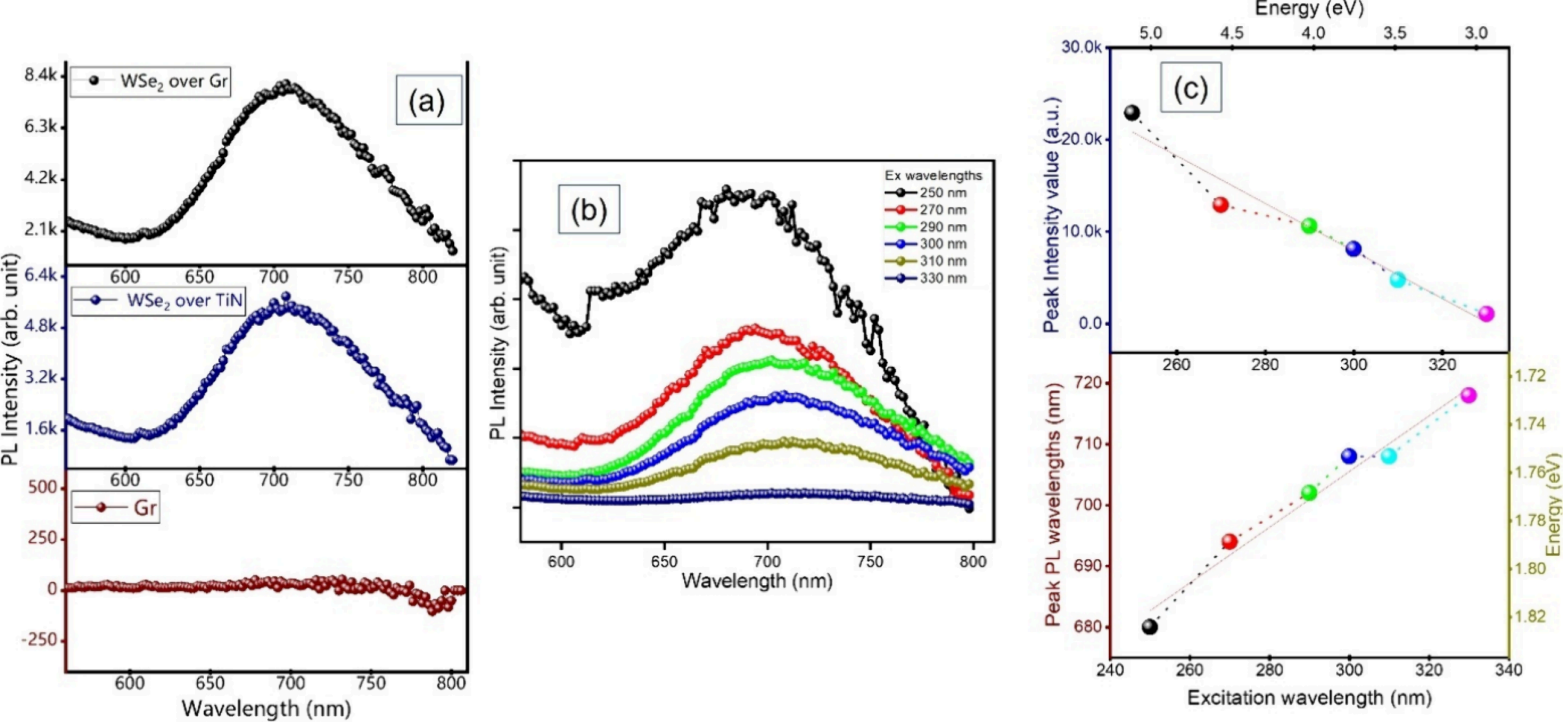
(a) PL scans for graphene, electrodeposited WSe_2_ (600 s) on TiN and on graphene using a Xe lamp (450 W) as an excitation light source with the excitation wavelength of 300 nm. (b) Emission mapping of WSe_2_ over graphene for different excitation wavelengths. (c) Plot of excitation wavelength versus peak intensity (top) and peak PL wavelength (bottom).

Furthermore, Figure 6b presents excitation-wavelength-dependent PL measurements of the WSe_2_ film over the graphene layer. The peaks of the emission and the corresponding PL intensity are extracted from Figure 6b and plotted in Figure 6c. Both the peak intensity and peak wavelength have a clear dependence on the excitation energy. The peak energy shifts from 1.82 to 1.72 eV with decreasing excitation energy. This is slightly higher than the typical WSe_2_ monolayer/few-layer emission at 1.65 eV. This behaviour suggests that higher excitation energy can activate additional electronic states beyond the K-point, modifying the emission characteristics.^61^ The broad nature of the PL emission (>100 nm) can be attributed to defect-assisted recombination and localized excitonic states. The electrodeposition process introduces grain boundaries, vacancies, and localized trap states, all of which contribute to spectral broadening.^62^ Additionally, inhomogeneous strain distribution and local potential fluctuations can result in a range of recombination energies, further widening the emission spectrum.^63,64^ The clear excitation around the bandgap energy nevertheless testifies to the semiconducting properties of the electrodeposited WSe_2_, demonstrating the first reported PL emission from electrodeposited WSe_2_.

## 4 Conclusions

We report the growth of large-area WSe_2_ films on graphene via electrodeposition using WSeCl_4_ as an in-house synthesized single-source precursor. The electrochemical behavior of the precursor was investigated via cyclic voltammetry studies, and a reduction potential around −1.4 V was found to be suitable for the electrodeposition of WSe_2_. Raman spectroscopy was used to observe the signature of the electrodeposited film, confirming the successful growth of WSe_2_ and the suitability of graphene to be used as an electrode in electrodepositing this material. AFM measurements confirm that the electrodeposited WSe_2_ on graphene exhibits a few-layer thickness of 4 nm with significantly higher uniformity than that of previously reported electrodeposited films. The W:Se composition ratio in the material is found to be 1:1.83 based on the XPS measurement, and TEM imaging demonstrated the well-ordered stacks of the 2D layers and the crystal structure of WSe_2_ on graphene. We then performed photoluminescence measurements to study the light emission and semiconductor properties of the electrodeposited films, which showed broad emission centred at 710 nm, demonstrating the first PL emission from an electrodeposited transition metal dichalcogenide.

Electrodeposition is a cost-effective and industry-compatible method that has the potential to scale the deposition of transition metal dichalcogenides and their heterostructures with graphene into wafer sizes. Our future works will involve adjusting the electrodeposition and annealing parameters to increase the crystal domain size to improve the PL efficiency of WSe_2_ and develop p-doped electronic devices based on this material. Furthermore, this work paves the way toward utilizing electrodeposition to stack multiple TMDs, including MoS_2_, WS_2_, and WSe_2_ over graphene at wafer scales for electronic and optoelectronic applications.

## Supplementary Material



## Notes

Experiment details for graphene (Raman) and WSe_2_ (Raman, AFM and WDS) (PDF)
