# Supplementary material for: Large-Area Electrodeposited WSe2 over Graphene Electrodes for Optoelectronics
Source: ACS Appl Nano Mater. 2025 May 16;8(21):10842–50. doi: 10.1021/acsanm.4c07346 (PMC12131223; doi:10.1021/acsanm.4c07346)
Supplement: Supplementary file 1 [file an4c07346_si_001.pdf]

Supporting Information

Large-Area Electrodeposited WSe_2 over Graphene Electrodes for Optoelectronics

Jiapei Zhang[1], Shibin Thomas[2], Ahmad Nizamuddin Muhammad Mustafa[3,5], Victoria Greenacre[2], Nikolay Zhelev[2], Syeda Ramsha Ali[1], Yisong Han[4], Shaokai Song[1], Hongwei Zhang[1], Aiden Graham[1], Nema M. Abdelazim[1], Sami Ramadan[3], Richard Beanland[4], Gillian Reid[2], Philip N Bartlett[2], Kees de Groot[1], Yasir J Noori[1 *]

[1]School of Electronics and Computer Science, University of Southampton, Southampton SO17 1BJ, United Kingdom

[2]School of Chemistry and Chemical Engineering, University of Southampton, Southampton SO17 1BJ, United Kingdom

[3]Department of Materials, Imperial College London, London SW7 2AZ, United Kingdom

[4]Department of Physics, University of Warwick, Coventry CV4 7AL, United Kingdom

[5]FTKEK, Universiti Teknikal Malaysia Melaka, 76100 Malacca, Malaysia

[*]Authors to whom any correspondence should be addressed.

E-mail: y.j.noori@southampton.ac.uk

Keywords: electrodeposition, 2D materials, TMD, tungsten selenide, WSe_2, graphene, electrode, heterostructure

Supplementary figures

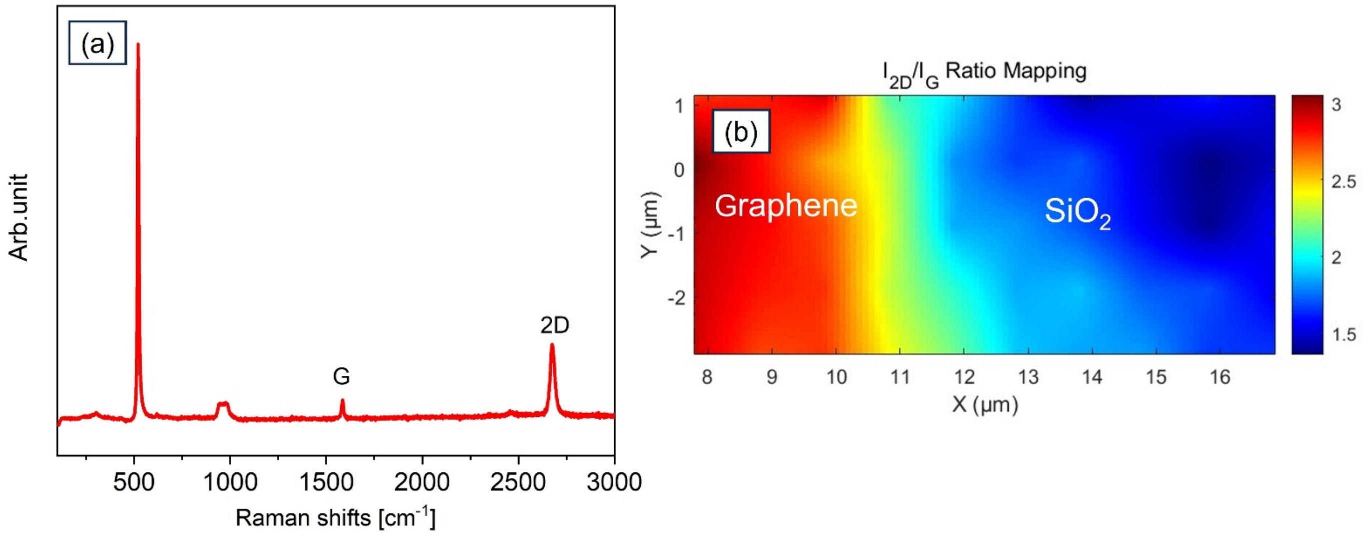

Figure S1. Raman results of graphene for (a) full range Raman spectrum of graphene, and (b) I_{2D}/I_G ratio mapping of graphene substrate where left red area is graphene over SiO_2 and right blue area is SiO_2 substrate.

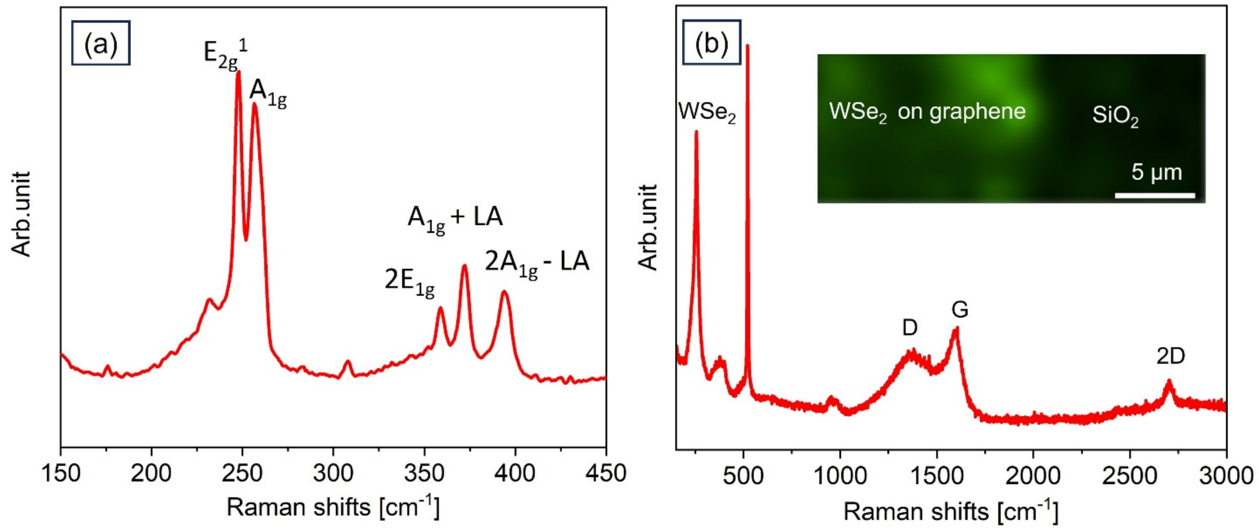

Figure S2. Ramn results of a commercial crystal reference WSe_2 and electrodeposited WSe_2 over graphene for (a) Raman spectrum of commercial reference WSe_2 highlighting major E^1_{2g} and A_{1g} peaks, and three minor peaks between 350 and 400 cm^{-1}. (b) Full range Raman spectrum of both graphene and WSe_2 after electrodeposition and annealing, highlighting major peak of WSe_2 (256.7 cm^{-1}), and three peaks of graphene for D (1373.0 cm^{-1}), G (1606.7 cm^{-1}) and 2D (2704.0 cm^{-1}), respectively. The inset image is 2D peak Raman mapping.

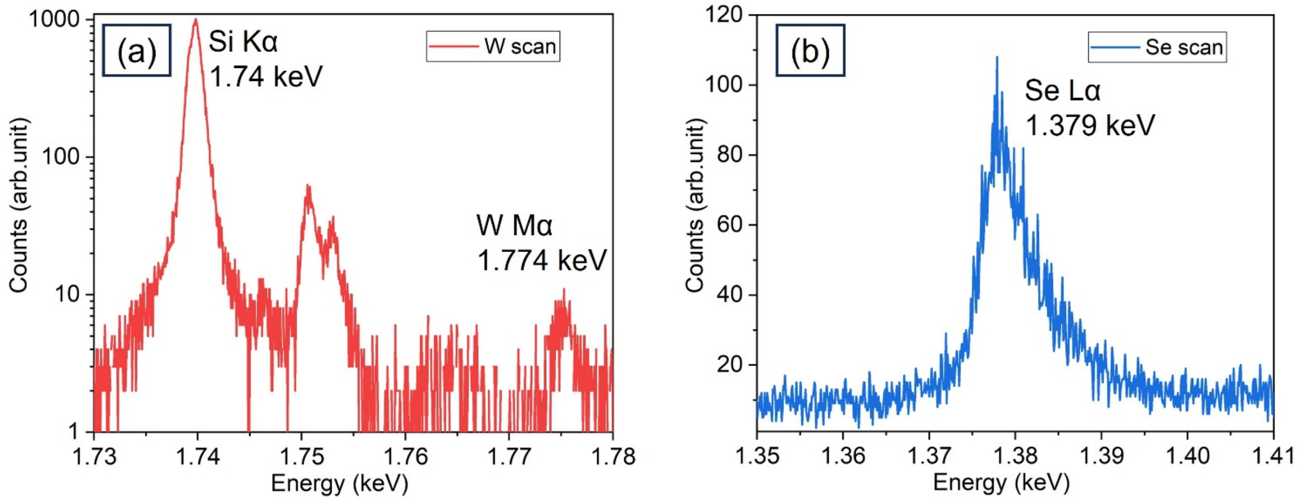

Figure S3. WDS spectrum of electrodeposited WSe_2 for (a) W scan highlighting Si (Kα) peak (1.74 keV) and W (Mα) peak (1.774 keV) and (b) Se scan highlighting Se (Lα) peak (1.379 keV).

XPS Data Treatment

The XPS spectra were acquired using a ThermoFisher XPS system and the data was acquired and analysed using the Thermo Avantage software (v 5.9925). Background baseline subtraction was performed with Thermo Avantage by subtracting point a background average within the plotted energy spectra. The average was taken from 0.5 eV bins, corresponding to 10 data points. The W and Se peaks were fitted as doublet in the software, fixing the peaks' intensity and FWHM to maintain the area ratios expected for the fitted peaks. The targeted goodness of fit was $Chi^2 < 2$.
